# Fabrication and Performance Evaluation of NiMOF@MGO-Modified Polysulfone Membranes for Heavy Metal Removal from Wastewater

**DOI:** 10.3390/polym18010117

**Published:** 2025-12-31

**Authors:** Javad Hashemibeni, Asif Jamil, Asta Bronusiene, Hesam Seifi, Arvydas Palevicius, Giedrius Janusas

**Affiliations:** 1 Department of Mechanical Engineering, Faculty of Mechanical Engineering and Design, Kaunas University of Technology, Studentų 56, LT-51424 Kaunas, Lithuania; javad.hashemibeni@ktu.edu (J.H.); muhammad.jamil@ktu.lt (A.J.); arvydas.paleviciu@ktu.lt (A.P.); 2 Department of Characterization of Materials Structure, Center for Physical Sciences and Technology (FTMC), Sauletekio Av. 3, LT-10257 Vilnius, Lithuania; asta.bronusiene@ftmc.lt; 3 Department of Chemical Engineering, Membrane Science and Technology Research Center, Gachsaran Branch, Islamic Azad University, Gachsaran P.O. Box 75818-63876, Iran; hesam.saifi64@gmail.com

**Keywords:** mixed matrix membrane, polysulfone, magnetic graphene oxide, MOF, nickel, water purification

## Abstract

This work presents a detailed analysis of polysulfone (PSF) based mixed matrix membranes (MMMs) modified with NiMOF@MGO for water purification. Magnetic iron oxide nanoparticles were synthesized and incorporated into the NiMOF@GO framework, with successful formation confirmed by FT-IR, XRD, BET, TGA, and SEM analyses. Membranes were prepared via phase inversion and modified with varying NiMOF@MGO contents. SEM, AFM, and contact angle analyses demonstrated enhanced membrane hydrophilicity with increasing MOF concentration, reducing the contact angle from 59.74° (0.05 wt%) to 49.70° (0.2 wt%). The highest flux of 117.85 L/m^2^·h was observed for the PMM-0.2 membrane. Heavy metal removal was most efficient at pH 6, with the PMM-0.1 membrane achieving 95.97% and 95.92% rejection for Pb^2+^ and Cu^2+^, respectively. In oil-water separation, PMM-0.1 exhibited optimal performance, with a water flux of 45.84 L/m^2^·h. Antifouling tests showed the PMM-0.2 membrane had the highest flux recovery of 85.97%, indicating improved fouling resistance. Overall, incorporation of NiMOF@MGO significantly enhanced membrane hydrophilicity, flux, selectivity and antifouling performance, demonstrating its potential for advanced water purification applications.

## 1. Introduction

In recent years, water pollution driven by rapid urbanization and industrialization has emerged as a critical environmental issue, primarily due to the discharge of heavy metals that contaminate water resources and aquatic ecosystems [1]. Even at trace concentrations, heavy metals are highly toxic and tend to bioaccumulate within both aquatic and terrestrial food chains. For example, exposure to lead ions adversely affects human health by impairing the cardiovascular system, kidneys, and brain, while mercury exposure has been linked to severe immune and neurological dysfunctions [2]. Over the past decades, numerous conventional methods have been developed to remove heavy metals from wastewater [3]. However, these traditional water treatment approaches are often insufficient for complete removal, thereby necessitating the development of more advanced technologies such as membrane filtration. Compared with conventional separation and desalination methods (e.g., distillation), membrane-based processes offer several advantages, including compactness, robustness, scalability, and improved energy efficiency [4]. Consequently, membrane separation has become a cornerstone in purification technologies [5], accounting for up to 53% of global processes for producing purified water. Its wide applicability in treating brackish water, wastewater, and seawater desalination for potable reuse stems from its operational simplicity, chemical-free nature, cost-effectiveness, absence of phase change, high efficiency, and strong removal capacity [6]. Acting as selective barriers, membranes are considered environmentally benign technologies capable of eliminating a wide variety of pollutants from water streams [7,8]. The application of reverse osmosis, nanofiltration, and mixed matrix membranes (MMMs) in the separation of heavy metals has been thoroughly investigated and discussed in prior studies [9,10]. Furthermore, advancements in materials science have enabled the discovery of novel building blocks and polymers, facilitating the design of next-generation separation membranes that meet key criteria: (1) uniform and well-defined pore size, (2) narrow pore size distribution, (3) ultrathin active layers, and (4) enhanced permeability [11,12].

Metal–organic frameworks (MOFs) are crystalline porous polymers formed through the coordination of metal ions with organic linkers. Their highly ordered and porous architectures impart exceptional stability, low density, and a remarkably large surface area [13]. In addition, the versatile synthetic routes available for MOFs enable precise control over pore size distribution, tunability of pore dimensions, and the incorporation of diverse surface functionalities. These unique attributes make MOFs highly promising for a broad spectrum of applications, particularly in separation processes [14]. Although MOFs inherently possess favorable physical and chemical properties for such applications, these characteristics can be further enhanced through various modification strategies [15]. In this context, the development of MOF-based composites with nanoparticles, such as iron oxide (Fe_3_O_4_) and silica, has been extensively reported and applied in separation technologies [16,17]. In this study, a nickel-based MOF (Ni-MOF) was selected as the filler material owing to its strong affinity for heavy metal ions and proven potential in water purification applications [18]. Ni-MOF exhibits a high specific surface area, good hydrophilicity, excellent thermal and cycling stability, and superior chemical resistance in aqueous environments compared with other commonly studied MOFs that often undergo partial degradation [19]. Furthermore, its tunable porosity and strong compatibility with polymer matrices facilitate uniform dispersion within the membrane, enhancing permeability and selective removal of toxic metal ions [20]. Moreover, the limited stability and partially inaccessible pores of MOFs restrict their full utilization. Integrating MOFs with two-dimensional (2D) materials can yield multifunctional hybrid structures that combine the advantages of both components, offering a promising route for developing advanced functional materials [21].

The aim of this research is to fabricate and characterize polysulfone (PSf) membranes modified with NiMOF@MGO for water purification. Therefore, first, magnetic nanoparticles are synthesized and combined with NiMOF@GO, and then MMMs are produced with the help of synthesized nanoparticles and used for water purification in membrane-based filtration system.

## 2. Experimental Procedures

### 2.1. Materials

Merck provided the following list of chemicals: sodium acetate (C_2_H_3_NaO_2_), ferric chloride (FeCl_3_), ethylene glycol (C_2_H_6_O_2_), terephthalic acid (C_8_H_6_O_4_) (TA), *N,N* dimethylformamide (DMF), nickel nitrate (Ni(NO_3_)_2_), ethanol (C_2_H_6_O), sulphuric acid (H_2_SO_4_), potassium permanganate (KMnO_4_), hydrogen peroxide (H_2_O_2_), tetraethyl orthosilicate (TEOS), lead nitrate (Pb(NO_3_)_2_), copper nitrate (Cu(NO_3_)_2_), solvent *N*-methyl-2-pyrrolidone (NMP > 95%), polyvinyl pyrrolidone (PVP > 97%), ethanol (C_2_H_5_OH), and toluene (C_7_H_8_). Following that, Sigma-Aldrich (Darmstadt, Germany) supplied bovine serum albumin (BSA), and BASF supplied the polysulfone polymer (PSF).

### 2.2. Synthesis of NiMOF@MGO Nanoparticles

The synthesis was performed in multiple steps. Fe_3_O_4_@SiO_2_ nanoparticles were prepared according to the method reported in the literature [22,23]. Graphene oxide (GO) was synthesized following the procedure described in [24,25]. Subsequently, Ni-based metal–organic framework (NiMOF) was obtained using the protocol outlined in [26].

In the last and final step, the NiMOF@GO nanoparticles were functionalized with Fe_3_O_4_ magnetic nanoparticles by ultrasonically dispersing 50 mg of NiMOF@GO in 4 mL of DMF (35 kHz) for two hours. Six milliliters of DMF were used to suspend 40 milligrams of FeO_4_@SiO_2_ nanoparticles in a separate container. The two mixtures were combined and constantly swirled for six hours. An external magnet was then used to separate the NiMOF@MGO hybrid, which was then washed three times with 50 mL of toluene before being dried for storage [26].

### 2.3. Preparation of MMMs

**Figure 1:**
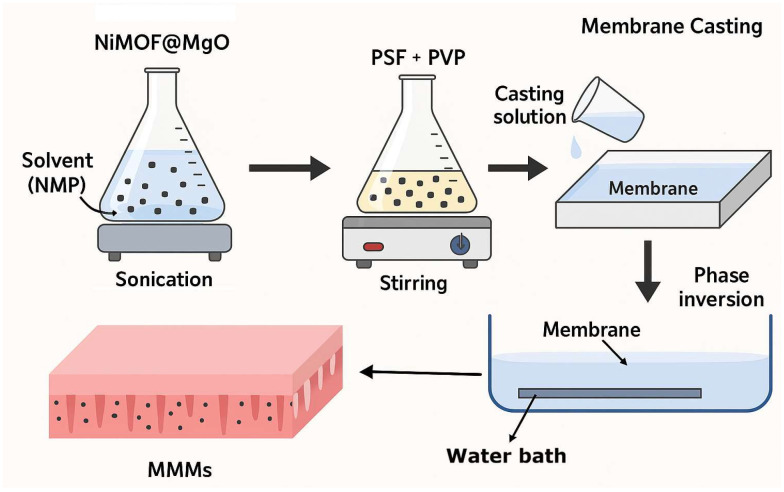
The schematic illustration of membrane developments steps.

The MMMs were developed using PSF as a base polymer and NiMOF@MGO nanoparticles as filler via phase inversion method as depicted in Figure 1. The membrane-forming solution was prepared by first dissolving 0.5 wt% PVP in 82 wt% NMP under magnetic stirring for 1 h. Subsequently, 17.5 wt% PSF was introduced gradually and mixed until a homogeneous solution was obtained. The mixture was then transferred to an Erlenmeyer flask equipped with a magnetic bar and stirred continuously for one day. The solution was ultrasonically degassed at room temperature for a further 24 h to eliminate trapped air bubbles [27]. The polymer solution was uniformly applied to a 20 × 20 cm glass plate and placed in a distilled water bath at 25 °C to promote phase inversion [28]. Then, it was placed in separate distilled water bath for a day, until the remaining solvent was removed, and then stored in distilled water. To modify the membrane with NiMOF@MGO nanoparticles, all the above steps were performed and different concentrations of NiMOF@MGO nanoparticles were added to the polymer solution. Table 1 shows the conditions for the fabrication of MMMs.

### 2.4. Characterization of Developed Materials

To investigate functional groups, an FT-IR spectrometer (VERTEX 70, Bruker, Billerica, MA, USA) was used. An atomic force microscope was used to analyze the surface as well as the structural characteristics of the membranes (AFM, JPK NanoWizard II, JPK Instruments, Berlin, Germany). The water contact angle of the developed membranes was measured with an optical contact angle goniometer (CAG-20 SE, Jikan, Tehran, Iran). A field emission scanning electron microscopy (SEM, S-3400N, Hitachi, Tokyo, Japan) was used to investigate the morphologies of the nanoparticles and membranes. The thermal characteristics of the nanoparticles were evaluated by thermogravimetric analysis (TGA, SDT-Q600, TA Instruments, New Castle, DE, USA).

### 2.5. Evaluation of Membrane Filtration Performance

**Figure 2:**
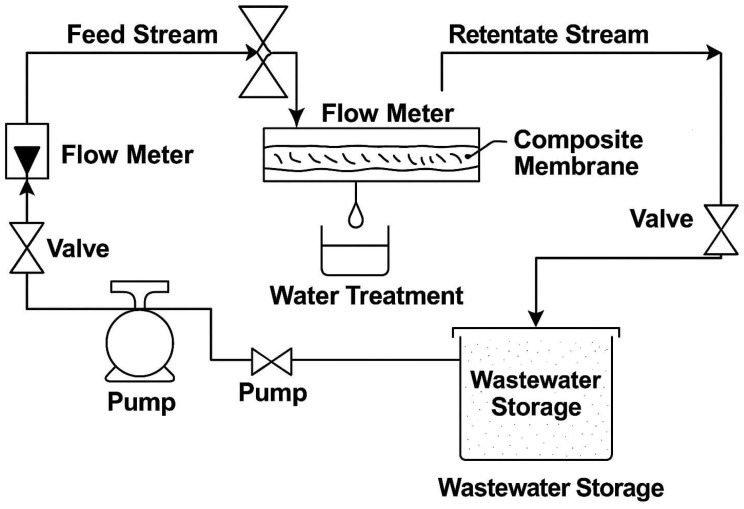
Schematic representation the membrane system for investigating the performance of developed composite membranes.

The membrane setup used to evaluate the performance of the developed membranes as shown in Figure 2, with an effective area of 14.45 cm^2^. The filtration performance was evaluated at 6 bar pressure, 3 L/min flow rate, for 1 h at ambient temperature. Aqueous heavy metal solutions were prepared by dissolving Pb(NO_3_)_2_, Cu(NO_3_)_2_, and Na_2_HAsO_4_·7H_2_O to a final concentration of 50 mg/L in deionized water. Oil with the properties described in Table 2 was used to prepare the solution for filtration.

Crude oil and distilled water were combined in a 1:1 (*v*/*v*) ratio with sodium dodecyl sulfate (SDS, wt%) as a surfactant. The mixture was vigorously stirred with a mechanical stirrer for 10 min to form a stable oil-in-water emulsion [29]. Water flux through the pristine as well as MMMs was calculated using Equations (1) and (2) [30].

(1) $$J=\frac{\Delta V}{A_{m}\cdot \Delta t}$$

(2) $$R=\left(1-\frac{C_{p}}{C_{f}}\right)\times 100$$

Here, *A_m_* represents the effective membrane area, Δ*V* is the volume of water that permeates through the membrane, Δ*t* is the filtration time, and Δ*P* denotes the applied pressure difference [30]. Similarly, *C_p_* and *C_f_* represent the concentrations of lead and copper in the permeate and in the feed solution, respectively. Heavy metal concentrations in the samples were determined using an atomic absorption spectrometer (280FS AA, Agilent, Santa Clara, CA, USA), while oil concentrations were measured with a UV–VIS spectrophotometer (UV-1900i, Shimadzu, Kyoto, Japan).

### 2.6. Antifouling Performance of Developed Membranes

The antifouling performance of the developed membranes was assessed using bovine serum albumin (BSA) filtration. Following preliminary measurements of the pure water flux, the membranes were filtered with the BSA solution for 120 min at 6 bar, and the permeate flux was noted. After the membranes were washed with deionized water, the flow of pure water through the cleaned membranes was measured, and Equation (4) was used to compute the flux recovery ratio (FRR). To assess membrane fouling further, the total fouling ratio (TFR), reversible fouling ratio (RFR), and irreversible fouling ratio (IFR) were calculated using the relevant Equations (5)–(7) [31].

(3) $$FRR(\%)=\frac{J_{W2}}{J_{W1}}\times 100$$

(4) $$RFR(\%)=(\frac{J_{W2}-J_{AS}}{J_{W1}})\times 100$$

(5) $$FR(\%)=(\frac{J_{W1}-J_{W2}}{J_{W1}})\times 100$$

(6) $$TFR(\%)=RFR(\%)+IFR(\%)=(\frac{J_{W1}-J_{AS}}{J_{W1}})\times 100$$

where $J_{W1}$ and $J_{W2}$ denote the permeate fluxes measured before and after membrane fouling, respectively, while $J_{AS}$ represents the flux recorded during the fouling process.

## 3. Results

The sections consist of two parts, one for the synthesized nanoparticles and the other for the developed membranes.

### 3.1. Characterization of Nanoparticles

**Figure 3:**
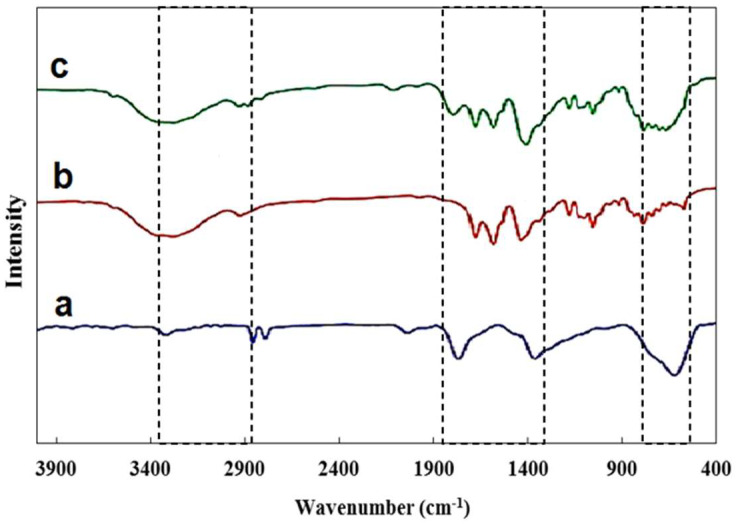
FT-IR spectrum (a) Fe_3_O_4_@SiO (b) NiMOF@GO (c) NiMOF@MGO.

The FT-IR spectra of developed composite are shown in Figure 3. The existence of magnetic graphene oxide is confirmed by characteristic peaks seen at about 1439 cm^−1^, 1676 cm^−1^, and 2862 cm^−1^, which correspond to C=C, C=O, and C–H functional groups [32]. The bands at 1025 cm^−1^ and 1218 cm^−1^ are attributed to C–O stretching vibrations in the alkoxy and epoxy groups of GO [33,34]. A broad band around 3350 cm^−1^ indicates O–H stretching in carboxylic groups [35].

Additional peaks further confirm the incorporation of NiMOF: the band at 560 cm^−1^ is assigned to Ni–O bonds, while those at 1232 cm^−1^, 1234 cm^−1^, and 1662 cm^−1^ correspond to C–O stretching, aromatic C–C, and C=O stretching vibrations, respectively [36]. Peaks at 611 cm^−1^ and 1784 cm^−1^ are associated with Fe–O and Si–O groups [23,37]. Furthermore, the signals at 539 cm^−1^ (Ni–O) and 614 cm^−1^ (Fe–O) provide strong evidence for the successful incorporation of NiMOF and Fe_3_O_4_@SiO_2_ into GO. Finally, the band at 1780 cm^−1^, assigned to Si–O, confirms effective silica coating [36,38].

**Figure 4:**
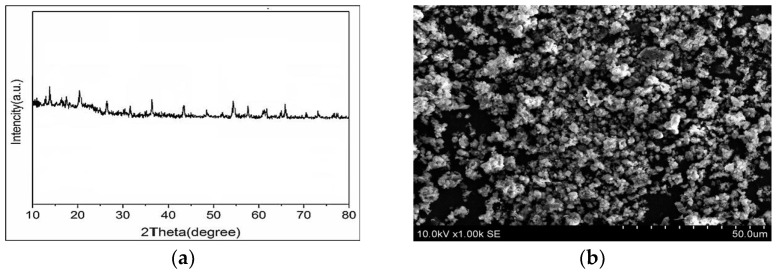
(**a**) XRD pattern, and (**b**) SEM image of NiMOF@MGO composite.

Figure 4a shows the X-ray diffraction pattern of NiMOF@MGO composite. As can be seen, Fe_3_O_4_ nanoparticles displayed unique diffraction peaks associated with the (1 1 1), (0 2 2), (1 1 3), (0 0 4), (2 2 4), (1 1 5), (0 4 4) and (5 5 3) planes [39,40,41]. Also, the peak appearing at 23° is attributed to the Si-O characteristic because of the magnetic silicate layer nanoparticles [42,43]. According to the X-ray diffraction pattern, weak peaks appeared at 2θ values around 11.4°, 13.1°, 17.8° and 24.9° which are consistent with the NiMOF structure [38], indicating the proper embedding of NiMOF on the GO surface.

SEM was employed to investigate the morphology, particle size, and surface characteristics of the NiMOF@MGO nanoparticles. As shown in Figure 4b, the nanoparticles exhibit a nearly uniform morphology with well-dispersed structures. The typical particle size of the monodispersed nanoparticles is in the range of 30–55 nm, indicating a narrow and uniform size distribution.

**Figure 5:**
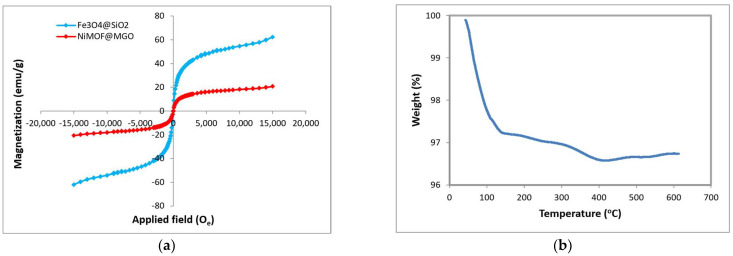
(**a**) VSM and (**b**) TGA of NiMOF@MGO composite.

A vibrating sample magnetometer (VSM) was used to assess magnetic characteristics of the nanoparticles at room temperature, and the resulting magnetic hysteresis loops, displayed as s-shaped curves, are shown in Figure 5a. The synthesized Fe_3_O_4_@SiO_2_ nanoparticles exhibit higher magnetic strength compared to the NiMOF@MGO composite nanoparticles. The saturation magnetization (Mₛ) of the composite was determined to be in the range of 3.75 emu/g. Therefore, the synthesized NiMOF@MGO composite has favorable magnetic properties.

Figure 5b presents the thermogravimetric analysis (TGA) of the NiMOF@MGO composite in the temperature range of 0–600 °C. An initial weight loss of approximately 2.75% around 100 °C is attributed to the evaporation of surface-adsorbed water as well as the release of water trapped within the bulk and internal sites of the composite. A further weight reduction of almost 3.47% occurs above 400 °C, which is mainly associated with the thermal degradation of organic components in the material [44].

### 3.2. Membrane Characterizations

**Figure 6:**
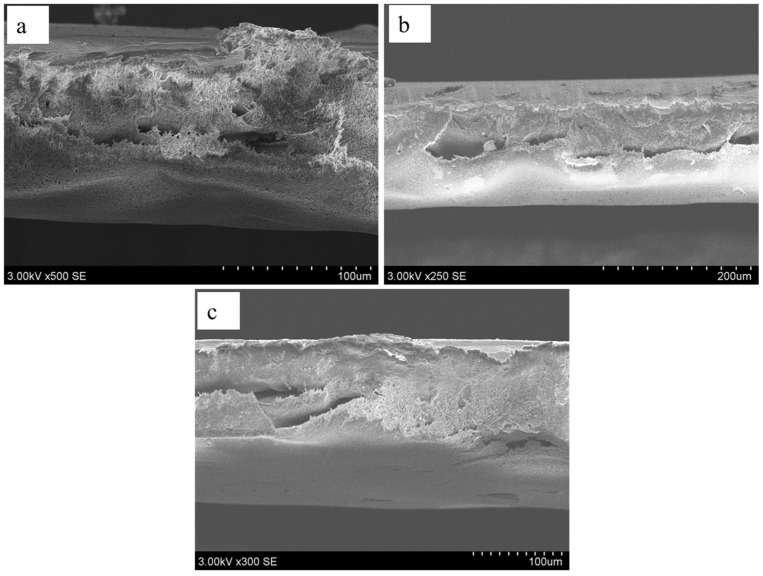
SEM cross-sections images of developed: (**a**) PSF, (**b**) PMM-0.1, and (**c**) PMM-0.2.

Figure 6 shows the cross-sectional view of the developed PSF and MMMs, highlighting the dominance of sponge-like morphology formed during phase inversion. This structure arises from controlled solvent–nonsolvent exchange, which ensures uniform polymer precipitation and prevents rapid demixing. Sponge-like networks, with their interconnected micropores and relatively uniform porosity, enhance mechanical stability and minimize the risk of collapse under operational conditions. Such morphology is advantageous for achieving balanced permeability and strength, as it provides adequate fluid transport pathways while maintaining structural integrity. Incorporation of NiMOF@GO further alters this morphology by acting as a pore-forming agent and disrupting polymer chain packing, resulting in increased porosity and surface roughness. SEM images confirm that NiMOF@GO-modified membranes exhibit a more open and irregular pore structure compared to pure PSF membranes. This enhanced roughness and porosity improve hydrophilicity, as MOF particles introduce polar functional groups that attract water molecules. Consequently, membranes containing NiMOF@GO demonstrate higher water flux and reduced fouling, making them highly suitable for filtration and separation applications [45,46].

**Figure 7:**
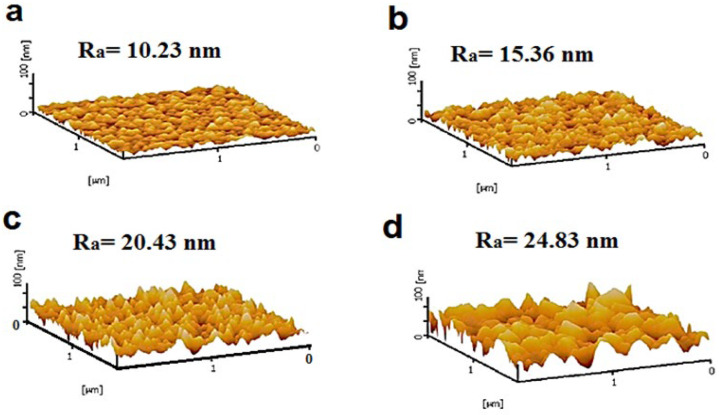
AFM images of membranes (**a**) PSF, (**b**) PMM-0.05, (**c**) PMM-0.1 and (**d**) PMM-0.2.

AFM was employed to characterize the surface topography of the fabricated membranes. The 3D AFM images of the developed membranes are presented in Figure 7, along with their corresponding average surface roughness (Ra) values. The results indicate that the incorporation of NiMOF@MGO increases the surface roughness of the MMMs compared to the pristine PSF membrane. Specifically, the PMM-0.2 membrane exhibits the highest Ra value of 24.83 nm. The observed increase in surface roughness correlates with the higher weight percentages of NiMOF@MGO in the polymer solutions used for membrane fabrication, which also increases the solution viscosity and the casting shear rate, both of which influence the membrane surface morphology. Higher-viscosity polymer solutions promote the formation of finer and more uniform pores, resulting in a rougher surface. This enhanced roughness contributes to increased membrane hydrophilicity and improved resistance to surface fouling [47,48].

The hydrophilicity of the fabricated membranes was evaluated using contact angle measurements. As shown in Table 3, incorporating NiMOF@MGO into the membranes significantly reduced the water contact angle, indicating enhanced hydrophilicity. Specifically, the membrane containing 0.05 wt% composite exhibited a contact angle of 59.74°, which decreased to 17.84° (a reduction of 49.70°) when the composite concentration was increased to 0.2 wt%. This trend confirms that higher NiMOF@MGO content increases membrane hydrophilicity, attributable to the presence of hydrophilic functional groups within the NiMOF@MGO composite [49,50].

**Figure 8:**
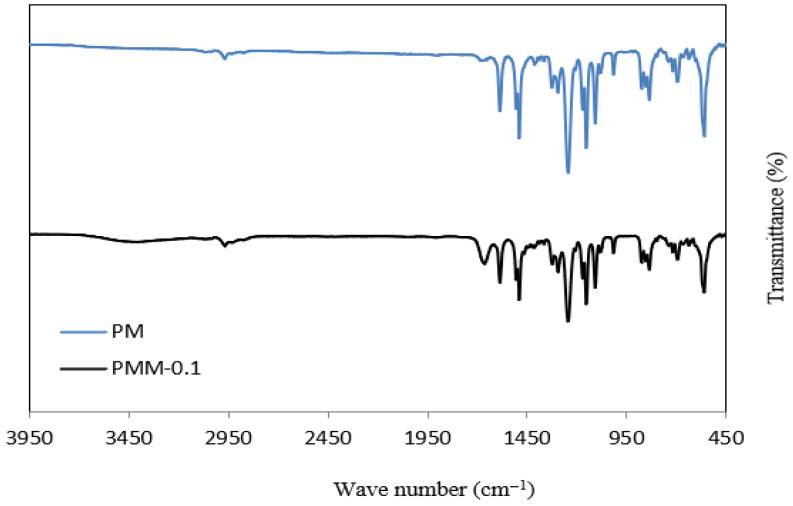
FT-IR Profiles of PSF and PMM-0.1 membranes.

The FT-IR spectra of the PSF and PMM-0.1 membranes are shown in Figure 8. PSF groups are the source of the distinctive peaks at 1160 cm^−1^, 1301 cm^−1^, and 1403 cm^−1^, which represent symmetric O=S=O stretching, asymmetric O=S=O stretching, and aromatic C=C ring stretching vibrations, respectively [51,52]. Peaks in the range of 832–1111 cm^−1^ are attributed to Si–O functional groups and covalent bonding [53]. O-H stretching vibrations are shown by a wide band between 3200 and 3400 cm^−1^, which validates the existence of hydroxyl groups. The peak at 1433 cm^−1^ is associated with water absorption and –CH_2_ stretching [54], while the band at 3006 cm^−1^ corresponds to aromatic C–H vibrations. Furthermore, the peaks at 1522 cm^−1^ and 1726 cm^−1^ confirm strong interactions between MGO and the NiMOF@MGO composite surface [55,56].

### 3.3. Results of Pure Water Flux Permeability of Deve006Coped MMMs

**Figure 9:**
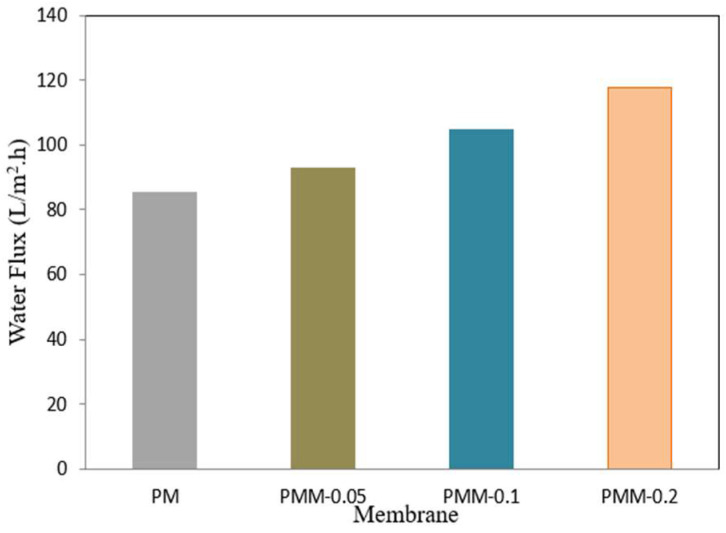
Permeation flux of pure water across developed membranes.

The pure water flux of the MMMs, measured during a 60 min period at a pressure of 6 bar and a flow velocity of 3 L/min, is shown in Figure 9. A key factor that sheds light on the hydrophilicity of the membranes is the water flow through the manufactured membranes [57,58]. When compared to the pristine PSF membrane, the MMMs incorporating NiMOF@MGO showed much greater water flow. The decreased hydrophilicity of the developed membrane is the reason for its comparatively low water flux (12.85 L/m^2^·h). PMM-0.2 showed the maximum water flux of 117.85 L/m^2^·h among the membranes under investigation, which is in line with its lower water contact angle and therefore higher hydrophilicity. The addition of NiMOF@MGO is responsible for the rise in water flow of these MMMs because it alters the average pore radius, increases surface microporosity, and improves membrane porosity, all of which promote increased water permeability [59].

### 3.4. Separation Performance of Developed Membranes

**Figure 10:**
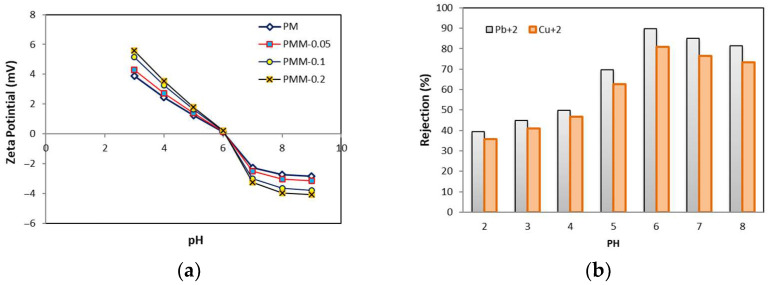
(**a**) Surface charge of composite membranes (**b**) Effect of pH on heavy metal rejection using PMM-0.05 membrane.

Figure 10 illustrates the effect of pH on the removal of heavy metals, specifically lead and copper. The membrane filtration experiments were conducted at a pressure of 6 bar for 60 min at ambient temperature. Solution pH is a critical parameter influencing both heavy metal separation and the surface charge of the membranes. In membrane processes, pH is often adjusted to enhance heavy metal removal efficiency [60,61]. The pH of the aqueous solution affects the surface charge of the MMMs, which becomes negative or positive at pH values above or below the membrane’s point of zero charge (pH_pzc_). Figure 10a shows the surface charge of the membranes at different pH values. The results indicate that the surface charge decreases from pH 2 to 6, with the pH_pzc_ observed at approximately pH 6. Consequently, electrostatic attraction between the membrane and heavy metal ions is expected to increase as the pH rises from 2 to 6. Experiments conducted over a pH range of 3 to 8 showed that the rejection of heavy metals, including lead, mercury, and arsenic, increased with increasing pH. The highest rejection was observed at pH 6, likely due to enhanced electrostatic interactions between the membrane surface and the heavy metal ions under mildly acidic conditions [62].

**Figure 11:**
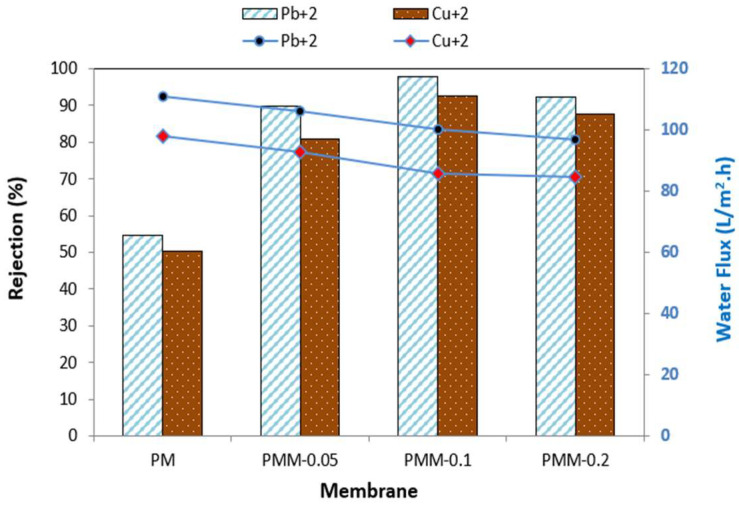
Heavy metal rejection and water flux across developed membranes.

Figure 11 presents the heavy metal rejection (Pb^2+^ and Cu^2+^) and water permeation flux of the developed membranes measured at pH 6, 6 bar pressure, for 60 min at ambient temperature. The MMMs modified with NiMOF@MGO nanoparticles exhibited significantly higher rejection compared to the pristine PSF membrane. This enhanced performance is primarily attributed to the size-exclusion (sieving) and Donnan rejection mechanisms [63]. Rejection is also influenced by the hydrated radius of the ions, which affects their ability to diffuse through the membrane pores. Since Pb^2+^ has a larger hydrated radius than Cu^2+^, its rejection percentage is higher [64]. The study further demonstrated that increasing the NiMOF@MGO content in the polysulfone polymer solution improves the removal efficiency of Pb^2+^ and Cu^2+^. However, at higher loadings (0.2 wt%, PMM-0.2), the increased viscosity of the dope solution promotes the formation of an asymmetric membrane structure, which can block surface pores and reduce water permeability, resulting in lower water flux compared to the pristine PSF membrane. The optimal performance was observed for the PMM-0.1 membrane, achieving heavy metal rejections of 95.97% for Pb^2+^ with a water flux of 45.83 L/m^2^·h, and 75.92% for Cu^2+^ with a water flux of 78.87 L/m^2^·h.

### 3.5. Investigation of the Antifouling Properties of Developed Membranes

An essential parameter for assessing antifouling capabilities of membranes is the flow recovery ratio (FRR). Based on BSA filtration experiments, the reversible fouling ratio (RFR), irreversible fouling ratio (IFR), total fouling ratio (TFR), and FRR were calculated to evaluate the fouling behavior of the developed membranes, as shown in Table 4. Better flux restoration is associated with higher FRR values, whereas more efficient overall fouling management is indicated by lower IFR values. Due to the enhanced hydrophilicity imparted by the NiMOF@MGO modification, all MMMs exhibited higher flux recovery compared to the pristine PSF membrane. Another important factor influencing fouling behavior is membrane surface roughness; a rougher membrane has more surface area accessible for foulant adsorption, which can affect water flow [65,66]. While irreversible fouling arises from the stable adsorption of foulants on the membrane surface or inside the pores, causing pore obstruction and decreased flow, reversible fouling is associated with contaminants that may be eliminated by washing [67]. The inclusion of NiMOF@MGO into the membranes successfully minimized irreversible fouling. With a flux recovery of 97.85%, PMM-0.2 had the greatest antifouling performance among the developed MMMs.

## 4. Conclusions

MMMs comprising PSF and NiMOF@MGO were fabricated and characterized analytically to study water purification performance. Incorporation of NiMOF@MGO significantly enhanced membrane hydrophilicity, as evidenced by the decrease in contact angle from 59.74° for 0.05 wt% composite to 17.84° (49.70°) for 0.2 wt%. When compared to the pristine PSF membrane, the MMMs showed a greater pure water flow, with the highest flux observed for the PMM-0.2 membrane (117.85 L/m^2^·h). Heavy metal removal was most effective at pH 6, with the PSF membrane demonstrating superior rejection compared to MMMs. The PMM-0.1 membrane achieved the best heavy metal rejection, with 95.97% (83.45 L/m^2^·h) for Pb^2+^ and 92.75% (87.78 L/m^2^·h) for Cu^2+^. For antifouling performance, the PMM-0.2 membrane exhibited the highest flux recovery at 97.85%, highlighting the beneficial effect of Ni-MOF@MGO incorporation on membrane stability and resistance to fouling.

These results indicate that incorporating NiMOF@MGO into the PSF membrane enhances its separation efficiency, antifouling behavior, and degradation performance, making it well-suited for industrial wastewater treatment and heavy metal removal applications.

## Figures and Tables

**Table 1:** Dope solution composition.

Membrane	PSF (%W_t_)	PVP (%W_t_)	NMP (%W_t_)	NiMOF/MGO (%W_t_)	Viscosity @ 25 °C (cp)
PM	17.50	0.50	82	0	7431
PMM-0.05	17.50	0.50	82	0.05	7719
PMM-0.1	17.50	0.50	82	0.1	7940
PMM-0.2	17.50	0.50	82	0.2	8211

**Table 2:** Oil properties.

SN	Property	Value
1	Specific gravity	0.8632
2	API	36.4
3	Sulfur content	1.41 wt%
4	Mercaptan content	19 ppm
5	Kinematic viscosity	3.95 mm^2^/s

**Table 3:** Contact angle measurements of developed membranes.

SN	Membrane Code	Contact Angle
1	PM	67.54°
2	PMM-0.05	59.74°
3	PMM-0.1	54.46°
4	PMM-0.2	49.70°

**Table 4:** Antifouling properties of developed membranes.

Membrane	RFR (%)	IFR (%)	TFR (%)	FRR (%)
PM	25	35	60	65
PMM-0.05	42	33	78	80
PMM-0.1	60	28	85	98
PMM-0.2	52	30	83	85

## Data Availability

The raw data supporting the conclusions of this article will be made available by the authors on request.

## Abbreviations

The following abbreviations are used in this manuscript:

MMM	Mixed Matrix Membrane
MOF	Metal–Organic Frameworks
PSf	Polysulfone
GO	Graphene Oxide
FT-IR	Fourier Transform Infrared Spectrometer
AFM	Atomic Force Microscope
CAG	Contact Angle Goniometer
SEM	Scanning Electron Microscopy
EDX	Energy-Dispersive X-ray Spectroscopy
TGA	Thermogravimetric Analysis
VSM	Vibrating Sample Magnetometer
BSA	Bovine Serum Albumin
FRR	Flux Recovery Ratio
TFR	Total Fouling Ratio
RFR	Reversible Fouling Ratio
IFR	Irreversible Fouling Ratio
Ra	Average Surface Roughness
